# Adult Neurogenesis Is Regulated by the Endocannabinoid and Kisspeptin Systems

**DOI:** 10.3390/ijms26093977

**Published:** 2025-04-23

**Authors:** Marianna Marino, Paola Di Pietro, Raffaella D’Auria, Martina Lombardi, Grazia Maria Giovanna Pastorino, Jacopo Troisi, Francesca Felicia Operto, Albino Carrizzo, Carmine Vecchione, Andrea Viggiano, Rosaria Meccariello, Antonietta Santoro

**Affiliations:** 1 Department of Medicine, Surgery and Dentistry “Scuola Medica Salernitana”, University of Salerno, 84081 Baronissi, Italy; mamarino@unisa.it (M.M.); pdipietro@unisa.it (P.D.P.); radauria@unisa.it (R.D.); marlombardi@unisa.it (M.L.); graziapastorino@gmail.com (G.M.G.P.); jtroisi@unisa.it (J.T.); acarrizzo@unisa.it (A.C.); cvecchione@unisa.it (C.V.); aviggiano@unisa.it (A.V.); 2 Child and Adolescent Neuropsychiatry Unit, San Giovanni di Dio Ruggi d’Aragona Hospital, 84131 Salerno, Italy; opertofrancesca@gmail.com; 3 Theoreo S.r.l. Montecorvino Pugliano, 84090 Salerno, Italy; 4 Department of Health Sciences, School of Medicine, University Magna Grecia of Catanzaro, 88100 Catanzaro, Italy; 5 Vascular Physiopathology Unit, IRCCS Neuromed Mediterranean Neurological Institute, 86077 Pozzilli, Italy; 6 Department of Medical, Motor and Wellness Sciences, University of Naples Parthenope, 80133 Napoli, Italy; rosaria.meccariello@uniparthenope.it

**Keywords:** adult neurogenesis, rat hippocampus, kisspeptin system, endocannabinoid system, TRPV1

## Abstract

Neurogenesis is considered the most robust form of plasticity in the adult brain. To better decipher this process, we evaluated the potential crosstalk of Kisspeptin and Endocannabinoid Systems (KPS and ECS, respectively) on hippocampal neurogenesis. Male adolescent rats were exposed to kisspeptin-10 (KP10) and the endocannabinoid anandamide (AEA) administered alone or in combination with the type 1 cannabinoid receptor (CB1R) antagonist SR141716A. The expression of Kiss1 and Kisspeptin receptor (Kiss1R) has been characterized for the first time in rat hippocampus together with the expression of the CB1R and the Transient Receptor Potential Vanilloid 1 ion channel receptor (TRPV1). Results show that both systems inhibit neurogenesis by reducing the extracellular signal-regulated kinase (ERK) signaling. Despite little differences in the expression of Kiss1R and CB1R, TRPV1 is enhanced by both KP10 and AEA treatments, suggesting TRPV1 as a common thread. KP10 administration reduces CB1R expression in the dentate gyrus, while AEA does not. KPS, unlike ECS, promotes the expression of estrogen receptor α (ER-α) and glyceraldehyde-3-phosphate dehydrogenase (GAPDH), also upregulating sirtuin 1 (SIRT1), brain-derived-neurotrophic factor (BDNF), and c-Jun. These findings suggest that the interaction between ECS and KPS could be involved in the fine-tuning of neurogenesis, highlighting a novel role for KPS.

## 1. Introduction

For many years it was believed that the adult mammalian brain was a fixed post-mitotic structure, lacking the ability to generate new cells, as the neurogenic potential was thought to be limited to embryonic and early developmental stages [1]. This view was overturned in the 1960s, when pioneering research by Joseph Altman et al. presented evidence of neurogenesis in the hippocampus of adult rats [2] and guinea pigs [3]. In 1992, neural stem cells (NSCs) were isolated from adult mouse brains [4], and similar cells were later identified in the adult human hippocampus [5]. These landmark discoveries initiated a field of research examining the regulatory mechanisms and the functional significance of this process. While the effective existence of adult neurogenesis in humans has been a matter of wide debate, its occurrence is now well established [6,7,8]. To date, it is recognized that the hippocampus, a crucial component of the brain’s limbic system with a pivotal role in memory, spatial navigation, and learning functions, is a primary site for adult neurogenesis. Indeed, the latter seems to be limited to specific neurogenic niches within the brain, which include the subventricular zone (SVZ) of the lateral ventricles and the subgranular zone (SGZ) of the dentate gyrus (DG) within the hippocampus [9,10]. Unlike the rapid and widespread embryonic neurogenesis, adult neurogenesis is a relatively rare and finely regulated event. Though we are still far from fully deciphering the intricate network of regulators involved in this process, current understanding indicates that a plethora of intrinsic factors as well as modulatory systems play a role in shaping its rate and extent. These include, but are not limited to, trophic factors, cytokines, neurotransmitters, hormones, and environmental factors [11,12]. Indeed, extensive research is currently underway to further examine this process, as shedding light on the mechanisms underlying adult neurogenesis may provide crucial insights into neural plasticity dynamics with great potential for therapeutic implications [13]. From a clinical perspective, enhancing adult neurogenesis could be instrumental for replacing neurons lost in neurodegenerative disorders and supporting cognitive preservation throughout aging.

In recent years the endocannabinoid system (ECS) has gained attention as a potential key regulator of adult neurogenesis [14]. The ECS consists of endogenously produced, lipid-based signaling molecules known as endocannabinoids—primarily anandamide (AEA) and 2-arachidonoylglycerol (2-AG)—alongside their receptors and the enzymes that mediate their synthesis and degradation. The endocannabinoids primarily interact with G protein-coupled receptors (GPCRs), including the type 1 and type 2 cannabinoid receptors (CB1R and CB2R, respectively) [15]. While the CB2R can be primarily found in the immune cells, the CB1R is highly expressed in the central nervous system, especially in GABAergic interneurons and in some subpopulations of glutamatergic neurons. Moreover, its expression has been widely reported in NSCs and gradually decreases over the maturation of the newly formed neurons [14]. Nonetheless, other potential receptors for endocannabinoids have been identified beyond the core ECS, including the capsaicin-sensitive ion channel receptor, Transient Receptor Potential Vanilloid 1 (TRPV1), and the nuclear receptors known as Peroxisome Proliferator-Activated Receptors (PPARs) [16]. Within the brain, this system exhibits crucial modulatory roles, impacting functions ranging from synaptic plasticity to stress response as well as pain and emotional regulation, besides its neuroprotective and immunomodulatory effects [15,17]. Currently, extensive literature supports the involvement of the ECS in adult neurogenesis, where it has been shown to influence key stages such as the proliferation, differentiation, and survival of the newly formed neurons [14]. However, despite significant research efforts, the mechanistic aspects underlying these effects remain unclear, and findings in the literature have often yielded conflicting results. For instance, a significant reduction in hippocampal neurogenesis has been observed in CB1R-knockout mice [18], in line with other studies supporting a pro-neurogenic role of ECS, mediated by CB1R [19,20]. On the other hand, contrasting findings have been described in other studies indicating that AEA, through the CB1R, inhibited the differentiation of cortical neuron progenitors to mature neurons and decreased PC12 neuronal-like generation by decreasing extracellular signal-regulated kinase (ERK) activation, which is responsible for nerve growth factor action [21]. These discrepancies may be attributed to various factors, including differences in animal models, experimental design, and treatment selection. Moreover, a potential dose-dependent effect has been proposed, suggesting that cannabinoids may stimulate neurogenesis at low concentrations (up to 1 μmol/L) and have inhibitory effects at higher concentrations [22]. Notwithstanding, given the intrinsic complexity of the ECS and the numerous signaling pathways with which it interacts, it is reasonable to speculate that possible communications with other signaling systems may constitute additional points of modulation. Some evidence supports a potential cross-talk between the ECS and the Kisspeptin System (KPS) [23]. The latter is composed of kisspeptins, a family of neuropeptides originating from the *Kiss1* gene and binding to a GPCR known as the Kiss1 receptor (Kiss1R) or GPR54. Kisspeptin (Kiss1L) and its receptor are primarily expressed in the hypothalamus, especially in neurons of the arcuate (ARC) nucleus and anteroventral periventricular nucleus [24]. These neurons play a crucial role in regulating the hypothalamic-pituitary-gonadal (HPG) axis, in which the KPS is involved by stimulating the release of the gonadotropin-releasing hormone (GnRH), influencing sex hormone production and fertility [25,26]. The presence of an active KPS has also been observed by in situ hybridization in several brain regions, such as the pons, midbrain, thalamus, hippocampus, amygdala, cortex, frontal cortex, and striatum [27]. In the hippocampus, *GPR54* mRNA was found in the granule cell layer of DG and scarcely detectable in the pyramidal cells of Cornus Ammonis 1 (CA1) and 3 (CA3) [27]. Moreover, activation of GPR54 with kisspeptin-10 (KP10) caused a extracellular signal-regulated kinases (ERK 1/2)-dependent increase of the synaptic potentiation in the granule cells of the DG [28], highlighting that Kiss1L could act as an autocrine factor regulating synaptic transmission within the hippocampus-hypothalamic circuit [29,30]. It has already been observed that the CB1R is expressed in various subpopulations of kisspeptin neurons [31]. Moreover, our research group demonstrated that the ECS and the KPS interact to modulate the HPG axis, with this interaction specifically involving the CB1R, suggesting a coordinated regulatory role over reproductive functions [23]. Beyond reproductive effects, however, emerging research suggests that the KPS may extend its influence to other processes, ranging from emotional regulation to energy homeostasis [32,33,34], while little is known about its potential neurogenic implications.

In light of the above, the present study aimed to investigate the role of the KPS and ECS, and their potential crosstalk, on hippocampal adult neurogenesis through in vivo experiments on male adolescent rats. Animals were administered with KP10, the smallest active peptide of Kiss1 precursor, or AEA, individually and alongside the CB1R antagonist SR141716A (SR). Morpho-functional evaluations of the KPS and ECS have been carried out within the hippocampus following the KPS and ECS stimulation; the potential mechanism of action and the effects on key markers of neuronal proliferation and differentiation have also been studied.

## 2. Results

### 2.1. Effects of KP10, AEA, and AEA+SR Treatment on Adult Neurogenesis

**Figure 1:**
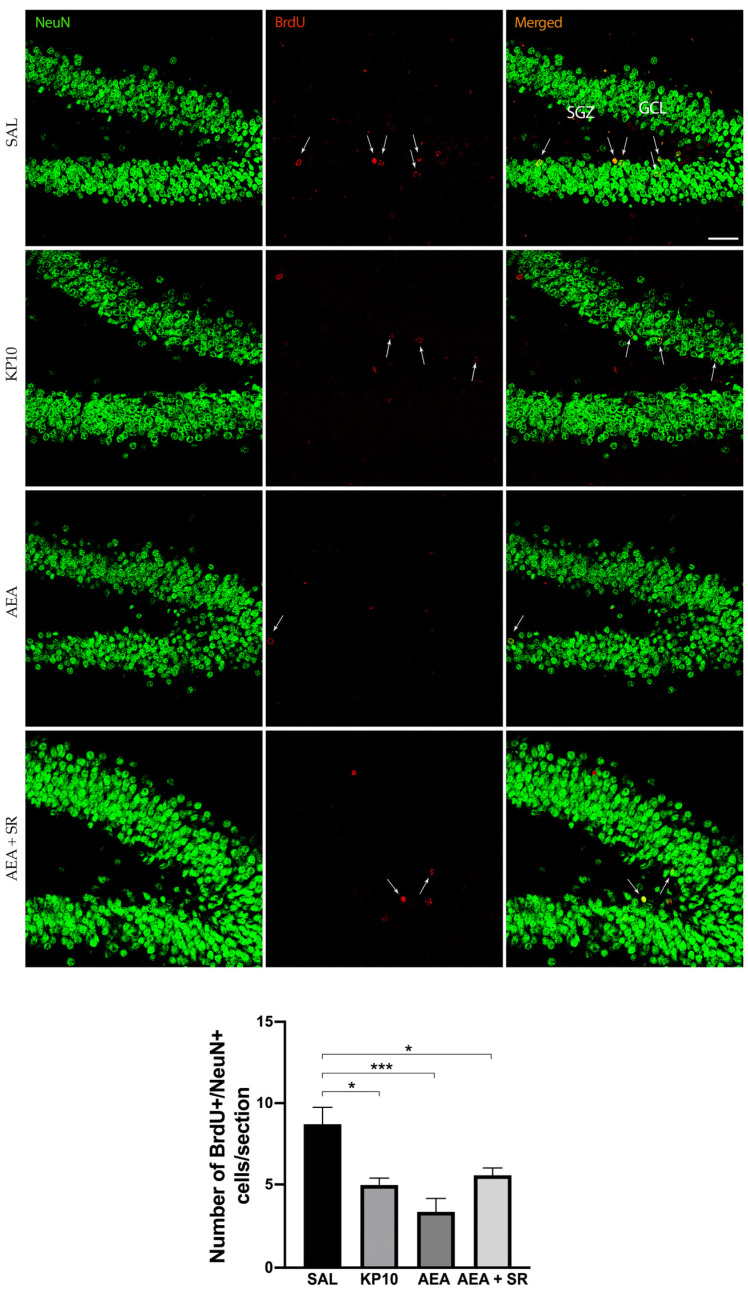
Effects of KP10, AEA, and AEA + SR treatments on adult neurogenesis. Representative images showing double IF staining for BrdU (red) and NeuN (green) in rat DG of control (SAL), kisspeptin (KP10), anandamide (AEA), and AEA plus SR141716A (AEA + SR) treated animals (*n* = 5). Arrows indicate BrdU/NeuN colocalization. Scale bar = 50 μm. SGZ, subgranular zone; GCL, granule cell layer. The number of BrdU-positive cells out of the total number of NeuN cells/section is given in the histogram below as mean ± SEM. *, *p* < 0.05, ***, *p* < 0.001.

To study the effects of KPS and ECS stimulation on adult neurogenesis, rats were treated with AEA or KP10. Doses were chosen based on previously published work [23]. The number of BrdU+ cells in the SGZ of the hippocampal DG was quantified in both treated and untreated rats. Results (Figure 1) indicate that both KP10 and AEA treatments significantly reduced the number of newborn neurons compared to the control group, and SR141716 (SR) scarcely attenuated the AEA-induced reduction of BrdU-positive cells.

### 2.2. KPS Expression in CA3 and DG

**Figure 2:**
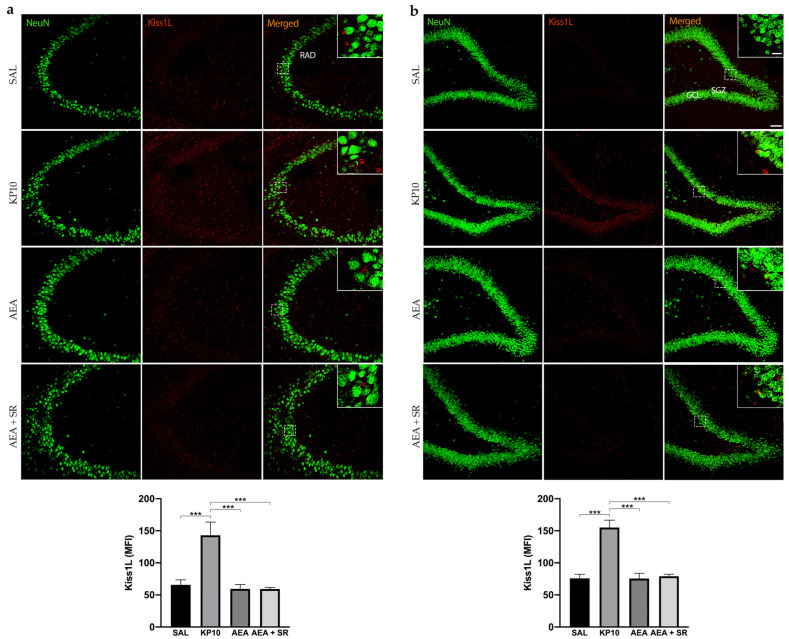
Expression and distribution of Kiss1L in the hippocampus. Representative images showing double IF staining for NeuN (green) and Kiss1L (red) expression in rat CA3 (**a**) and DG (**b**) of control (SAL), kisspeptin (KP10), anandamide (AEA), and SR141716 plus AEA-treated animals (*n* = 5 for SAL, AEA, and AEA + SR; *n* = 4 for KP10). Scale bar = 75 μm. High magnification insets show partial colocalization between Kiss1L and NeuN in the dentate gyrus. Scale bar = 15 μm. Histograms show the mean fluorescence intensity (MFI) of Kiss1L expression in hippocampal CA3 (**a**) and DG (**b**) of treated and untreated groups. RAD, stratum radiatum; SGZ, subgranular zone; GCL, granule cell layer. Data are expressed as mean ± SEM. ***, *p* < 0.001 vs. controls.

To date, no direct evidence has confirmed the expression of Kiss1L and Kiss1R proteins in mammals’ hippocampus. To provide information on this issue and also elucidate the above finding, we characterized the expression of the KPS in this brain region and then analyzed whether the treatment with KP10, AEA, and AEA + SR could influence the expression of both Kiss1L and Kiss1R. As shown in Figure 2a, Kiss1L is diffusely distributed throughout the CA3 region, extending into the stratum radiatum (RAD). In the hippocampal DG, Kiss1L is present not only in the GCL but also along the DG molecular layers (Figure 2b). Interestingly, in both hippocampal regions, Kiss1L higher magnification panels highlight that Kiss1L colocalizes only partially with NeuN staining, suggesting a possible localization of the protein in other resident cell populations. KP10 treatment leads to a marked increase in Kiss1L expression compared to controls. Likewise, the increase of Kiss1L by KP10 treatment was statistically significant compared to AEA and AEA + SR treatments.

**Figure 3:**
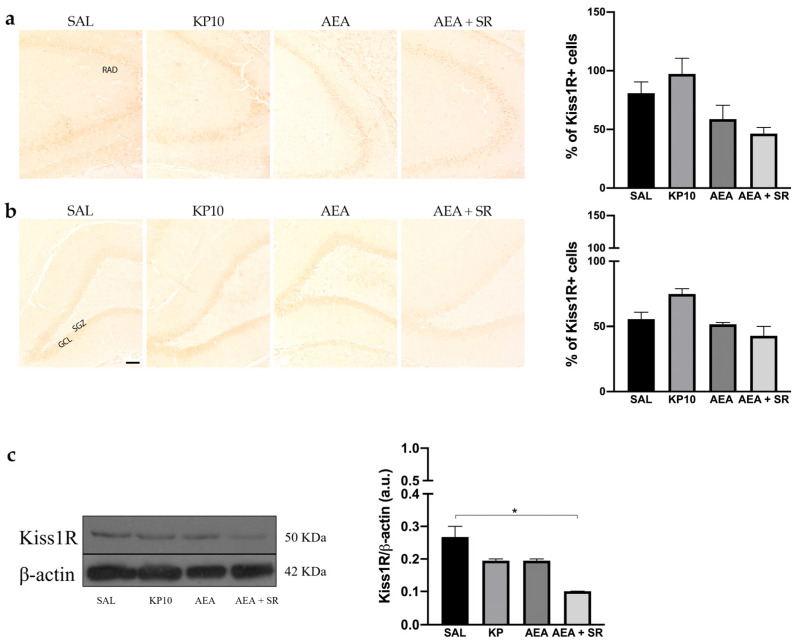
Expression and distribution of Kiss1R in hippocampus. Representative images showing IHC staining in rat CA3 (**a**) and DG (**b**) for Kiss1R expression (brown) of control (SAL), kisspeptin (KP10), anandamide (AEA), and AEA plus SR141716-treated animals (AEA + SR). RAD, stratum radiatum; SGZ, subgranular zone; GCL, granule cell layer (*n* = 4 for each group). Scale bar = 100 μm. Histograms below the images show the percentage of Kiss1R-positive compared to controls. (**c**) Representative immunoblot of kiss1R expression in controls and treated animals. β-actin was used as a loading control (*n* = 3 for each group). Data are expressed as mean ± SEM. * *p* < 0.05.

The expression of Kiss1R was analyzed in the hippocampus by immunohistochemistry (IHC) and western blot (WB). In all experimental groups, Kiss1R localized at the cell membrane but not in neuronal projections. Regarding the CA3 region, Kiss1R was mainly expressed in the stratum pyramidal (SP) (Figure 3a). In the DG, Kiss1R was distributed along the GCL, with some positive immunoreactivity in the hilus (HI) (Figure 3b). Even though it seems that a slight modulation is present in KP10, AEA, and AEA + SR treatment groups, these differences are not statistically significant. WB analysis corroborated the presence of Kiss1R in the hippocampus, showing a signal at about 50 KDa (Figure 3c). A slight, non-significant decrease was observed in the KP10- and AEA-treated groups compared to the controls, whereas only the decrease observed in the AEA + SR treatment group was statistically significant compared to the other groups.

### 2.3. CB1 and TRPV1 Expression in DG and CA3

**Figure 4:**
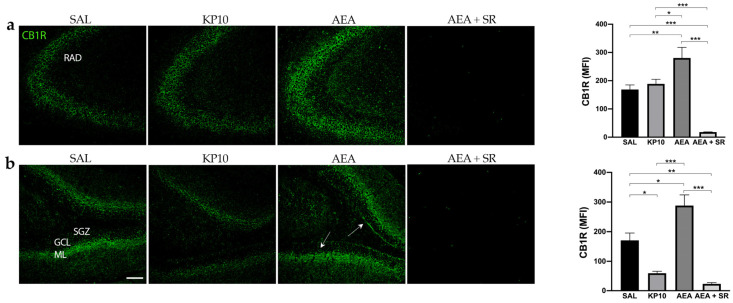
Expression and distribution of CB1R in the hippocampus: Representative images showing IF staining for CB1R expression (green) in rat CA3 (**a**) and DG (**b**) of control (SAL); kisspeptin (KP10); anandamide (AEA); and AEA plus SR141716 (SR)-treated animals (*n* = 4 for SAL, KP10, and AEA groups; *n* = 5 for AEA + SR group). Arrows indicate CB1R localization. Scale bar = 100 μm. Histograms show the mean fluorescence intensity (MFI) values of CB1R expression in hippocampal CA3 and DG. RAD, stratum radiatum; SGZ, subgranular zone; GCL, granule cell layer; ML, molecular layer. Data are expressed as mean ± SEM. *, *p* < 0.05; **, *p* < 0.01; and ***, *p* < 0.001.

To gain further insight into the possible interplay between KPS and ECS, we investigated the CB1R expression in the same brain regions. As shown in Figure 4a, most CB1R expression in CA3 was observed along neuronal projections. Notably, CB1R was also found in the fibers of pyramidal neurons of the CA3. As expected, CB1R expression was higher in the AEA-treated group, and this increase was completely reversed in the presence of SR. On the contrary, KP10 treatment did not cause any variation in the CB1R expression within the CA3. Furthermore, CB1R immunoreactivity was observed throughout the DG in all groups, specifically in the GCL, HI, and inner molecular layer (IML) (Figure 4b). In this region, the treatment with KP10 significantly reduced CB1R expression, whereas AEA induced it. The AEA induction of CB1R expression was completely reverted by treatment with the CB1 receptor antagonist SR in both CA3 and DG.

**Figure 5:**
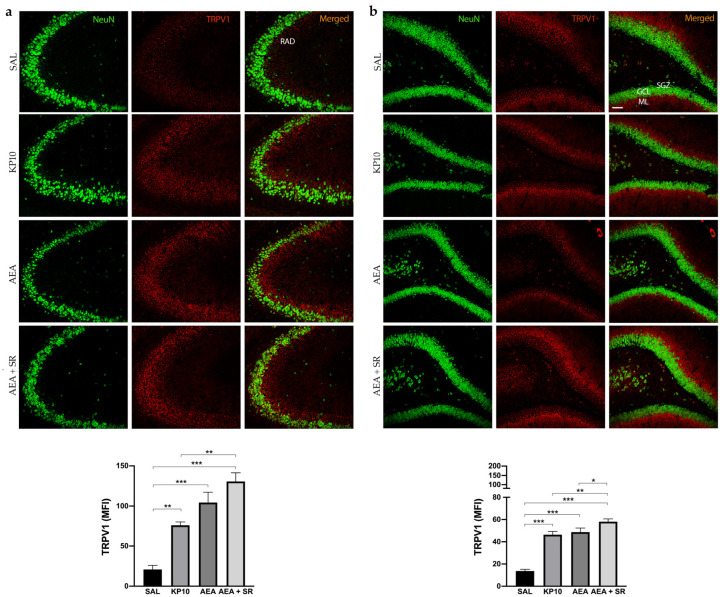
Expression and distribution of TRPV1 in the hippocampus. Representative images showing double IF staining for NeuN (green) and TRPV1 (red) expression in rat CA3 (**a**) and DG (**b**) of control (SAL), kisspeptin (KP10), anandamide (AEA), and AEA plus SR-treated animals (*n* = 5 for each group). RAD, stratum radiatum; SGZ, subgranular zone; GCL, granule cell layer; ML, molecular layer. Scale bar = 75 μm. Histograms show the mean fluorescence intensity (MFI) values of TRPV1 expression. Data are expressed as mean ± SEM. *, *p* < 0.05; **, *p* < 0.01; and ***, *p* < 0.001 vs. controls.

The above results indicated that KP10 treatment can alter the expression of the CB1R in an opposite way compared to AEA in the DG. Moreover, SR completely reversed the AEA-induced effect. To further investigate the involvement of other ECS receptors, we assessed TRPV1 expression across all treatment groups. Indeed, TRPV1 is widely expressed in the hippocampus, and it is well known to be a key ionotropic receptor within the ECS involved in neuronal differentiation and synaptic pruning [35]. As shown in Figure 5a, TRPV1 is strongly expressed at the cell membrane of the mature neurons (NeuN-positive cells) of the CA3 region. Additionally, its expression extends along neuronal projections. A similar pattern of expression was observed in the hippocampal DG (Figure 5b), where TRPV1 was detected in the HI and, more prominently, within the DG molecular layer (ML). Interestingly, in both regions, treatment with KP10, AEA, and AEA + SR resulted in a significant increase in TRPV1 expression (Figure 5a,b). These findings suggest a direct modulation of this receptor by both KP10 and AEA. Interestingly, the co-treatment with SR also induced TRPV1 expression markedly, failing to revert the AEA-mediated effect.

### 2.4. ECS and KPS Stimulation Reduce Adult Neurogenesis by Inhibiting ERK1/2 Signaling Pathway

**Figure 6:**
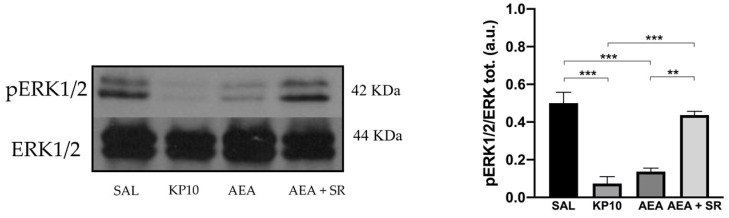
Reduced neurogenesis is mediated by the activation of the ERK1/2 protein in the rat hippocampus. Representative immunoblot (**left**) of phosphorylated ERK1/2 expression in control (SAL), kisspeptin (KP10), anandamide (AEA), and AEA plus SR of treated animals. β-actin was used as a loading control. Densitometric analysis (**right**) showing relative band intensity means (arbitrary units, a.u.) ± SEM (*n* = 3 for each group). **, *p* < 0.01; ***, *p* < 0.001.

To further characterize the molecular mechanisms underlying the observed effects, WB analysis was performed to evaluate the expression levels of the ERK1/2 signaling pathway [36]. The activation of ERK influences neural progenitor fate, promoting their proliferation [37]. Furthermore, ERK1/2 activity in the hippocampus is critical for facilitating Long Term Potentiation (LTP), which positively regulates neurogenesis and is essential for neuronal maturation, memory acquisition, learning, and neuronal activity [38,39]. Therefore, the level of phosphorylated ERK1/2 (pERK) was assessed across different treatment conditions. Results indicate a significant reduction in pERK1/2 activation in the KP10- and AEA-treated groups (Figure 6), and the effect induced by AEA was reverted by SR.

### 2.5. KP10 but Not AEA Induces Molecular Pathways Involved in Neuronal Differentiation

**Figure 7:**
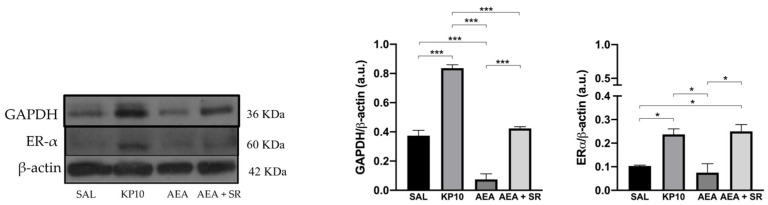
Modulation of neuronal differentiation markers in rat hippocampus. Representative immunoblot of GAPDH and Erα expressions in control (SAL), kisspeptin (KP10), anandamide (AEA), and AEA plus SR of treated animals. β-actin was used as a loading control. Densitometric analyses show relative band intensity means (arbitrary units, a.u.) ± SEM (*n* = 3 for each group). *, *p* < 0.05; and ***, *p* < 0.001.

After evaluating the influence of treatments on neuronal proliferation, the effects on neuronal differentiation were also considered. Given the established role of the estrogen receptor alpha (Erα) in mediating estradiol-induced effects on pathways crucial for inducing neuronal differentiation [40,41] the expression of Erα was assessed. As shown in Figure 7, both KP10 and AEA + SR treatments significantly upregulated Erα expression compared to controls, while AEA treatment resulted in a reduction of Erα expression, although this was not statistically significant. In addition, the expression of glyceraldehyde-3-phosphate dehydrogenase (GAPDH) was investigated. Although GAPDH is traditionally considered a housekeeping protein, recent studies have highlighted its role in neuritogenesis and the formation of neuronal processes [42]. As depicted in Figure 7, KP10 treatment led to a significant increase in GAPDH expression, while AEA treatment significantly reduced its expression. The AEA-induced reduction in GAPDH was partially reversed by SR, showing a trend toward upregulation. Interestingly, the effects induced by AEA and KP10 on GAPDH activity resulted statistically significant.

**Figure 8:**
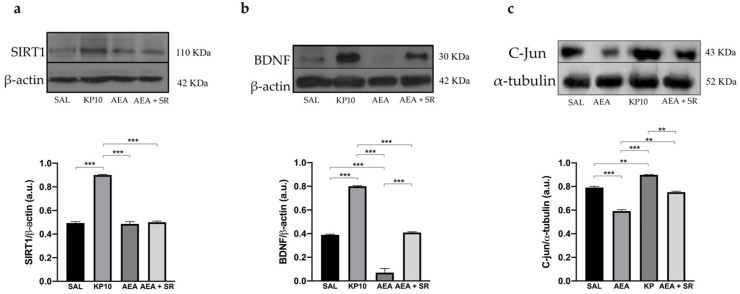
Modulation of SIRT1, BDNF, and c-Jun proteins. Representative immunoblot of SIRT1 (**a**), BDNF (**b**), and c-Jun (**c**) expressions in control (SAL), kisspeptin (KP10), anandamide (AEA), and AEA plus SR of treated animals. β-actin and β-tubulin were used as loading controls. Densitometric analyses show relative band intensity means (arbitrary units, a.u.) ± SEM (*n* = 3 for each group). **, *p* < 0.01; ***, *p* < 0.001.

Finally, the Sirtuin 1-Brain-Derived-Neurotrophic Factor (SIRT1-BDNF) signaling pathway, known to be essential for synapse formation and LTP, was evaluated [43,44]. Results show that KP10 treatment increases the expression of both SIRT1 and BDNF (Figure 8a,b). In contrast, AEA treatment significantly reduces BDNF expression, although SIRT1 levels remain unchanged. The co-treatment with SR and AEA partially reversed the AEA-induced effect on BDNF expression. In addition, statistical significance was observed between the group treated with AEA and that treated with KP. Finally, we evaluated the expression of c-Jun, a component of the AP-1 transcription factor whose increase positively correlates with neuronal activity. As shown in Figure 8c, KP10 enhances c-Jun expression while AEA treatment significantly reduces it. The AEA-induced effect is attenuated by AEA + SR. Altogether these findings suggest that the KPS and ECS can act on proteins regulating neuronal activity and differentiation.

## 3. Discussion

Adult neurogenesis, particularly in hippocampal DG, is a complex process regulated by various cellular and molecular factors. It includes cell proliferation, differentiation, maturation, and integration into existing neuronal circuits [45,46]. Among these factors, ECS has emerged as a key modulator, even though its exact role in adult neurogenesis remains debated, with conflicting data reported in literature [18,21,47]. Similarly, in addition to the recognized role of the KPS as gatekeeper of the HPG axis in mammals [25,26], Kiss1R mRNA has been found within the hippocampus [48], but its functional role is still unraveled. Aimed to better understand ECS’s role in neurogenesis and to explore its possible interaction with KPS, we first investigated the effects of in vivo modulation of the KPS and ECS by KP10 and AEA, focusing on AEA communications with the CB1R and related signaling molecules involved in proliferation and neuronal differentiation. Our findings are consistent with studies suggesting an inhibitory effect of the ECS on adult hippocampal neurogenesis via the CB1R [18,21]. Specifically, in our experimental conditions, AEA treatment reduces BrdU/NeuN-positive cells, and the ECS stimulation hampers ERK/MAPK activity, diminishing the phosphorylation of ERK1/2, crucial for the proliferation of neuronal multipotent progenitors [49]. Such results agree with the study by Rueda et al., who evidenced that AEA is able to decrease PC12 neuronal-like generation via CB1R-mediated inhibition of ERK activation [21]. Moreover, ERK1/2 activity in the hippocampus also modulates plastic events such as LTP, stimulating the proliferation of progenitor cells in the DG with a consequential long-term persistence of a larger population of surviving newborn cells [39].

Although we have described the presence of Kiss1 ligand and receptor proteins within the hippocampus, some limitations should be addressed. Concerning Kiss1R, it was evaluated by IHC and not IF. However, IHC is a well-established and equally valid technique for detecting and localizing specific antigens within tissue sections, allowing us to obtain information comparable to that of IF [50]. On the other hand, we did not provide information on the molecular weight of Kiss1L. This could be due to lower levels of Kiss1L in the hippocampus. Indeed, it is already documented that the hippocampus contains about 50–100 times lower Kiss1L mRNA than the hypothalamus, and this may explain why the peptide remained below detection in WB analysis [28,51]. In our experimental conditions, few newborn neurons (BrdU/NeuN-positive) have been detected by using a single dose of BrdU. However, this small number is in line with what has been described in other studies, and it is known that the levels of neurogenesis in adult mammalian brains are low compared to embryonic ones [52,53].

Notably, KP10 treatment caused a decrease of BrdU/NeuN-positive cells, as also observed in AEA-treated animals. Moreover, since KP10 significantly reduces CB1R in the DG, a functional crosstalk between the ECS and the KPS is also conceivable. Our results also show that in AEA-treated rats, the CB1R-induced effects are only partially lessened by the CB1R antagonist SR (rimonabant), suggesting that some effects might be CB1 independent. This is not surprising since previous findings suggest that SR can promote neurogenesis in CB1 knockout mice [18], acting as an inverse agonist rather than an antagonist [54,55]. On the other hand, in our experimental conditions, both KP10 and AEA increase the expression of TRPV1, proposing an additional signaling pathway in the KPS/ECS crosstalk. The possible involvement of the TRVP1 in neurogenesis is consistent with the findings of Jin et al., reporting that the neurogenesis-enhancing effect of SR was absent in TRPV1 knockout mice [18]. Currently, the mechanisms underlying the interaction between SR and TRPV1 remain uncharted; however, it is conceivable that this interaction could trigger a transient surge in calcium signaling, activating pathways linked to cell survival and neurogenesis [18,35]. From this point of view, our findings add evidence for TRPV1′s potential role in ECS-mediated effects on neurogenesis and show that TRPV1 may be the fil rouge for the action of both ECS and KPS in the hippocampus. This is supported by the fact that TRPV1 can be phosphorylated by multiple protein kinases, including PKA and PKC [56]. It is plausible that the activation, of the GPR54 receptor induces PKC activation which in turn amplifies TRPV1 activity [56,57,58]. In addition, it has been shown that intraplantar injection of kisspeptin in mice caused a robust increase in (Ser 800)-TRPV1 phosphorylation, an effect that could be mediated by GRP54 receptor activation [57]. Indeed, spontaneous calcium oscillations, induced by TRPV1 activation, may play an important role also in nervous system development, neural induction, axon guidance, growth cone morphology, migration, and proliferation [35,59,60,61,62]. Our results suggest that kisspeptins, through the increased expression (maybe activation) of the TRPV1, may reduce neurogenesis but promote neuronal differentiation. The possible involvement of the KPS in cell differentiation is consistent with previous data in tumors [63], spermatogonial cell lines [64], adipocytes [65], or osteoprogenitors [66]. Accordingly, we have shown that KP10 promotes the expression of key proteins such as ER-α, GAPDH, SIRT1, and BDNF involved in the formation of neuronal processes, neurite outgrowth, synaptic function, and plasticity in the DG and CA3 regions of the hippocampus [40,41,42,43,67]. Specifically, the observed upregulation of the c-Jun protein, a key component of the AP-1 transcription factor, corroborates that KP10 is able to stimulate neuronal activity [68].

As concerns ECS, a possible explanation for the obtained results indicating a reduction of SIRT1, BDNF, and c-Jun expressions is that CB1R stimulation in neurons could potentially elicit responses that hinder neuronal differentiation and neuronal activity [21,69]. Alternatively, KPS could be more active in promoting cell differentiation [70,71]. Present data once again confirm the link between the KPS and estrogen signaling in brain and peripheral tissues [23,72,73]. Indeed, nuclear estrogen signaling increases BDNF and SIRT1 levels within the hippocampus [74,75,76], and estrogen-related pathways are critical in neurogenesis [77,78] but also in modulating AEA tone by the transcriptional regulation of the fatty acid amide hydrolase (FAAH) [78,79]. In this respect, KPS signaling may represent the switch to promote estrogen aromatization and signaling in the brain (present data and previous observations) [23,80,81]. Taken together, this might have important therapeutic and pharmacological implications in neurodegenerative disease management.

ECS and KPS interplay is recognized as crucial in the regulation of reproduction at the hypothalamic level [23], therefore, given the potential connection between the hypothalamus and hippocampus via the hypothalamic-pituitary-hippocampal axis (HPH) [30], it could be critical to reveal the specific role of ECS and KPS at the hippocampus level. This is plausible, as the effects of hypothalamus-pituitary-derived GnRH, Luteinizing Hormone (LH), and Follicle-Stimulating Hormone (FSH) regulate neuro estradiol production in the hippocampus, thus promoting such processes of the cell cycle (proliferation and/or differentiation) crucial for the regulation of neurogenesis [30]. At present, only the mRNA for GPR54 has been identified within the hippocampus [48], thus, this is the first report demonstrating the existence of Kiss1L and Kiss1R proteins within this brain region. However, drawing a definitive conclusion regarding the impact of ECS and KPS on neurogenesis still remains complex. The named systems could impact adult neurogenesis at various levels across different stages. Concerning ECS, the decrease in the percentage of BrdU-positive cells expressing NeuN could be attributed also to the effects of non-neuronal cells. Indeed, endocannabinoids, including AEA, are released “on demand” by both neuronal and glial cells in the hippocampal neurogenic niche, and once produced, these lipids act as autocrine and/or paracrine ligands, binding both their target receptors and other non-metabotropic receptors such as TRPV1. Notably, AEA and other endocannabinoids produced in postsynaptic neurons can spread into the local environment and affect not only the corresponding presynaptic neurons but also the neighboring neurons, being able to potentially modulate all steps of neurogenesis [14,82]. Concerning Kiss1L, although we do not have direct evidence that it is secreted in the hippocampus, previous literature indicates that, in the hypothalamic ARC nucleus, there are neurons co-expressing kisspeptin, neurokinin B, and dynorphin A, which are responsible for the generation of GnRH pulses. These neuropeptides are stored in intracellular neurosecretory vesicles and are released via exocytosis [83,84]. In view of the above, it is possible that similar mechanisms may occur within the hippocampus. The pathways triggered by the release of Kiss1L and AEA in various target cells may be different, but all are implicated in the complex signaling leading multipotent progenitor cells into the SGZ of the DG to mature in adult-born granule cells and astrocytes. This may also account for the different modulation of Kiss1R and other proteins by KP10 and AEA that we observed in WB. Moreover, because drug administrations occurred within three weeks after a single BrdU dose, it is conceivable that our assessment of neurogenesis captured only the later stages of neurogenesis. Indeed, it is noteworthy that NeuN serves as a marker for postmitotic cells, encompassing both “typical” postmitotic neurons and newly generated postmitotic neurons, thus providing an underestimation of the spectrum of ECS- and KPS-induced effects in all stages of neurogenesis [18,85,86]. Despite that, it is worthwhile to mention that even if not definitely proved, our results suggest that the interplay between KPS and ECS seems to be unidirectional, with KPS influencing ECS rather than the reverse. Therefore, further investigations are needed to corroborate the aforementioned hypothesis and to understand the precise mechanisms governing such interactions.

The intricate nature of these systems represents a formidable challenge to unravel the molecular and cellular pathways involved in the generation and differentiation of new postnatal neurons and lay the bases for advancing neurogenesis-based treatments against neurological disorders.

## 4. Materials and Methods

### 4.1. Drugs and Antisera

*N*-Arachidonylethanolamine (AEA, sc-396321A), SR141716A (rimonabant, SR, *N*-(piperidino-1-yl)-5-(4-chlorophenyl)-1-(2,4dichlorophenyl)-4-methyl-pyrazole-3-carboxamide sc-205491A) and Metastin 45–54 of human origin (KP10, h-YNWNSFGLRF-NH_2_-sc-221883) were all provided by Santa Cruz Biotechnology (Dallas, TX, USA). AEA and SR were solubilized in ethanol (1%) at concentrations of 0.05 M and 0.01 M, respectively, while KP10 was solubilized in saline at a concentration of 7 × 10^−4^ M. Before administration, stock solutions were diluted 1:100 in saline and subsequently administered intraperitoneally. Table 1 shows the primary and secondary antisera and working conditions employed for WB, IF, and IHC analyses. The antibodies against Kiss1R [87,88] and Kiss1L [89,90] used in this study have been shown to reliably detect their respective targets in neural tissues.

### 4.2. Animal Studies

Male Wistar rats were purchased from Harlan Laboratories, Bresso, Italy (body weight 250–300 g). Upon arrival, forty peripubertal rats, 38 post-natal days (pnd), were housed in pairs for each cage and individually recognized through tail tags. Animals were randomly divided into four experimental groups (*n* = 10/experimental group): group 1, SAL (Control: 1% EtOH); group 2, KP10 (KP10: 0.1 mg/kg/bw); and group 3, AEA (AEA: 2 mg/kg/bw); group 4, AEA + SR (0.5 mg/kg/bw SR141716A + 2 mg/kg/bw AEA). Dosages were selected based on previous studies [23]. To avoid differences in stress conditions between rats injected with KP10 or AEA alone and those injected with AEA + SR, all rats received two intraperitoneal injections (twice a week) with a 30-min interval. Specifically, in the AEA + SR group, rats received SR injections first, followed by AEA administration. The other groups received a saline injection as the first dose, succeeded by the second one containing the appropriate drug.

**Figure 9:**
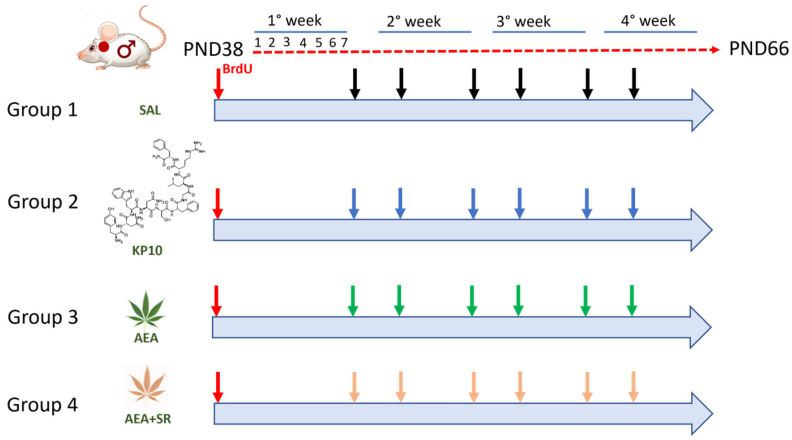
Diagram of the animal experimental protocol. Red arrows indicate treatment with BrdU; black arrows indicate controls (SAL); blue arrows indicate treatment with Kisspeptin10 (KP10); green arrows indicate treatment with Anandamide (AEA); and pink arrows indicate treatment with Anandamide plus SR141716A (AEA+SR).

After three weeks of treatment, rats were sacrificed by urethane (Sigma-Aldrich, Milano, Italy) (2 g/kg/bw). A flowchart of the experimental design is given in Figure 9. Following sacrifice, brains were removed and divided into two hemispheres, which were fixed overnight in a 4% paraformaldehyde (PFA) solution and randomly allocated for molecular and morphological assessments. In detail, hemispheres were fixed and transferred to 70% ethanol until they were embedded in paraffin and processed for IF, IHC, and WB analyses. Serial coronal sections were obtained along the bregma coordinates provided in the Paxinos and Watson rat brain atlas [91], ranging from −2.04 mm to −5.04 mm. In the contralateral hemisphere, the hippocampus was meticulously separated from the surrounding cerebral structures for subsequent WB analysis. From each experimental group, we chose the number of animals adequate to perform statistical analysis in all kinds of methodologies (IF, IHC, and WB). Only samples meeting quality control standards, such as sufficient sample quantity for protein extracts and high-quality slices with an intact hippocampal region free from structural damage and folds, were employed. During the experiment, rats were housed under standard temperature and humidity conditions with a 12 h light/12 h dark cycle and free access to standard fresh food and water. Experimental protocols were approved by the Ethical Committee of the University of Salerno and by the Italian Ministry of University and Research (authorization no. 66/2020-PR dated 29 January 2020).

All experimental procedures complied with the rules of the European Union Guide for the Care and Use of Laboratory Animals.

### 4.3. BrdU Treatment and Quantification

BrdU (5-bromo-2′-deoxyuridine), Thymidine analog (ab142567), was provided by Abcam and dissolved in NaCl at a concentration of 0.08 M. All the animals received a single intraperitoneal injection of 300 mg/kg/bw one week before the beginning of treatments [92,93]. To evaluate adult neurogenesis rats were sacrificed on day 28 after BrdU injection. The quantification of labeled cells was performed in six sections, spaced 420 µm apart, for each animal brain. These sections corresponded to coronal coordinates ranging from Bregma −2.04 mm to −5.04 mm, encompassing the entire hippocampus. Within each section, the number of immunoreactive cells stained with the anti-BrdU antibody, which co-localized with the neuronal nuclear antigen (NeuN) marker, was counted in both the upper and lower dentate granule cell layers (GCL) and the SGZ of the DG. Image acquisition was conducted using an inverted Leica laser-scanning confocal microscope TCS SP5 (Leica Microsystems, Wetzlar, Germany) at a magnification of 40×. Results were recorded as the average number of BrdU/neural nuclei (NeuN)-positive cells per section in each animal. Data were statistically analyzed as reported below.

### 4.4. Immunofluorescence

Serial 10 µm coronal sections of rat brain were sampled from bregma −2.04 to −5.04 mm. Sections were subsequently processed for immunohistochemical analysis of cells expressing NeuN, Kiss1R, Kiss1L, CB1R, TRPV1, and BrdU (details and working conditions in Table 1). Deparaffinized sections were treated for antigen retrieval with 10 mM citrate buffer (pH 6.0) and heated in a microwave for 40 min. Slides were cooled for 30 min in antigen retrieval buffers at room temperature, washed with PBS for 10 min, and then 0.1% Triton X-100 in PBS was added for 15 min for permeabilization. To analyze BrdU-positive cells, sections were incubated with 2 M HCL for 30 min at 37°C. After 3 washes with PBS (10 min/wash), slides were incubated in blocking solution (10% normal serum in PBS) for 1 h at RT, followed by overnight incubation with primary antibodies at 4 °C. Then, the sections were washed with PBS and incubated for 1 h with fluorescent dye-conjugated secondary antibodies. All images were acquired with an inverted Leica laser-scanning confocal microscope TCS SP5 (Leica Microsystems). Fluorochromes were detected using laser lines 488 and 543 nm. Double-stained images were obtained by sequential scanning for each channel to eliminate the crosstalk of chromophores and to ensure reliable co-localization. Images were then processed using ImageJ 1,54 d software.

### 4.5. Immunohistochemistry

Ten-micron-thick sections of the hippocampus were prepared and mounted on glass slides coated with poly-L-lysine. These sections underwent IHC analysis using the anti-Kiss1R antibody, with detailed information and working conditions provided in Table 1. The IHC protocol involved deparaffinization, non-enzymatic antigen retrieval, and permeabilization as described previously [23]. Subsequently, the slides were treated with a 3.6% hydrogen peroxide solution for 10 min to block endogenous peroxidases. Then, they were blocked with a solution containing 10% normal serum in PBS for 1 h at RT. After rinsing, the sections were incubated overnight at 4 °C with the anti-Kiss1R antibody. Further washes with PBS were performed, followed by incubation with Donkey anti-rabbit IgG Biotin for 1 h at RT. Next, slides were incubated with streptavidin-horseradish peroxidase (Vector Laboratories, Newark, CA, USA) for 1 h at RT. The immunoreaction signal was visualized using a substrate chromogen solution containing 3,3-diaminobenzidine tetrahydrochloride (DAB substrate kit, Vector Laboratories, Newark, CA, USA). Brightfield microscopy (Olympus SC180, Tokyo, Japan) at a 10× magnification was utilized for slide examination. The number of Kiss1R-positive cells within the DG and CA3 regions of the hippocampus was quantified in each section using ImageJ software. The average number of cells per animal was then calculated and employed for subsequent statistical analysis.

### 4.6. Western Blot

From brains fixed in 4 % PFA, the hippocampi were isolated and cut to obtain slices that were subsequently homogenized. Proteins were extracted using Formalin-Fixed Tissue Protein Extraction (FFPE kit purchased from Qiagen, Germantown, MD, USA), and concentrations were determined by Bio-Rad protein assay as described by Santoro et al., 2009 [54]. Equal amounts of protein extracts (20 μg) were boiled in Laemmli’s buffer, fractionated on SDS-PAGE, and then transferred to nitrocellulose membranes (Bio-Rad, Milan, Italy). Membranes were blocked in with 10% nonfat dry milk in PBS-T (0.05% Tween-20), washed in PBS-T, then incubated overnight at 4 °C with the following primary antibodies: anti-BDNF, anti-Kiss1R, anti-SIRT1, anti-ERK-1, anti-GAPDH, anti-pERK, anti-ER-α, and anti-c-Jun (details and working conditions in Table 1). Anti-β-actin and α-tubulin were used as loading controls. Blots were probed with mouse or rabbit horseradish peroxidase-conjugated secondary antibodies for 1 h at RT and then developed using Pierce ECL detection reagents (Thermo Scientific, Rockford, IL, USA) on X-ray films. Densitometry of bands was performed by using ImageJ software (NIH, Bethesda, MA, USA).

### 4.7. Statistical Analysis

Data were expressed as mean ± standard error of the mean (SEM). Comparison between multiple groups was assessed by one-way ANOVA followed by Bonferroni’s post hoc test. Statistical analysis was performed with GraphPad Prism software (version 5.0, GraphPad Software, San Diego, CA, USA). For all analyses, differences were considered statistically significant when *p* < 0.05.

## Figures and Tables

**Table 1:** List and working conditions of primary and secondary antisera used for protein analysis techniques.

IF Primary Antisera	Dilutions:
CB1 rabbit polyclonal IgG (AB23703, Abcam, Cambridge, UK)	1:100
Kiss1 (Kiss1L), clone 8H4.1 mouse monoclonal IgG1k (MABC60, Merck, Darmstadt, Germany)	1:50
NeuN rabbit monoclonal IgG (AB177487, Abcam, Cambridge, UK	1:500
BrdU, sheep polyclonal IgG-Proliferation Marker (AB1893, Abcam, Cambridge, UK)	1:50
TRPV1, mouse monoclonal [BS397] IgG2b (AB203103, Abcam, Cambridge, UK)	1:50
IF Secondary Antisera	
Horse anti-rabbit IgG Dylight 488 (DI-1088, Vector Laboratories, Kirtlington, Oxfordshire, UK)	1:200
Horse-anti-mouse IgG Dylight 549 (DI-2549, Vector Laboratories, Kirtlington, Oxfordshire, UK )	1:200
Donkey Anti-Sheep IgG H&L (Alexa Fluor^®^ 594; ab150180, Abcam, Cambridge, UK)	1:200
Donkey anti-Mouse IgG (H&L) Biotin (DkxMu-003-FBIO, ImmunoReagents, Raleigh, NC, USA)	1:100
Streptavidin, Alexa Fluor™ 647 Conjugate (S32357, Invitrogen, Thermo Fisher Scientific, Waltham, MA, USA)	1:100
IHC Primary Antisera	
Kiss1R rabbit polyclonal, C-terminally amidated peptide (BS-2501R, Bioss Antibodies, Woburn, MA, USA)	1:50
IHC Secondary Antisera	
Donkey-anti-rat IgG Biotin (DkxRt-003-FBIO, ImmunoReagents, Raleigh, NC, USA))	1:100
Horseradish Peroxidase Streptavidin (SA-5004-1, Vector Laboratories, Newark, CA, USA)	1:50
WB Primary Antisera	
Brain-Derived Neurotrophic Factor (BDNF) rabbit polyclonal IgG antibody (28205-1-AP, Proteintech, Rosemont, IL, USA)	1:1000
Phospho-p44/42 MAPK (Erk1/2) (Thr202/Tyr204) Rabbit mAb (4370 Cell signaling, Danvers, MA, USA)	1:3000
p44/42 MAPK (Erk1/2) Rabbit mAb (4695 Cell signaling, Danvers, MA, USA)	1:500
Glyceraldehyde-3-phosphate dehydrogenase (GAPDH) mouse monoclonal IgG2b antibody (60004-1IG, Proteintech, Rosemont, IL, USA)	1:1000
Anti-estrogen receptor alpha rabbit polyclonal IgG antibody (06-935, Merck, Darmstadt, Germany)	1:1000
SIRT-1 mouse monoclonal IgG1 antibody [19A7AB4] (ab110304, Abcam, Cambridge, UK)	1:3000
β-actin mouse monoclonal IgG1 antibody (sc-47778, Santa Cruz Biotechnology, Dallas, TX, USA)	1:1000
Kiss1R rabbit polyclonal (BS-2501R, Bioss Antibodies, USA)	1:1000
c-Jun (60A8) Rabbit mAb (#9165 Cell signaling, (Woburn, MA, USA))	1:1000
α-tubulin rabbit polyclonal IgG antibody (sc-5286; Santa Cruz Biotechnology, Dallas, TX, USA)	1:1000
WB Secondary Antisera	
Goat anti-Rabbit IgG, (H + L) HRP conjugate (AP307P, Merck, Darmstadt, Germany)	1:3000
Goat anti-Mouse IgG (H + L) HRP conjugate (AP308P, Merck, Darmstadt, Germany)	1:3000

## Data Availability

For inquiries about information, please write to the corresponding author.

## Abbreviations

The following abbreviations are used in this manuscript:

NSCs	Neural Stem Cells
SVZ	Subventricular Zone
SGZ	Subgranular Zone
ECS	Endocannabinoid System
AEA	Anandamide
2-AG	2-arachidonoylglycerol
GPCRs	G protein-coupled Receptors
CB1R	Type 1 Cannabinoid Receptor
CB2R	Type 2 Cannabinoid Receptor
TRPV1	Transient Receptor Potential Vanilloid 1
PPARs	Peroxisome Proliferator-Activated Receptors
KPS	Kisspeptin System
HPG	Hypothalamic-pituitary-gonadal Axis
GnRH	Gonadotropin-releasing Hormone
SR	SR141716A
KP10	Kisspeptin-10
WB	Western Blot
IF	Immunofluorescence
IHC	Immunohistochemistry
SAL	Control
BrdU	5-bromo-2′-deoxyuridine
NeuN	Neural Nuclear Antigen
GCL	Granule Cell Layer
DG	Dentate Gyrus
CA3	Cornus Ammonis 3
SEM	Standard Error of Mean
SP	Stratum Pyramidal
HI	Hilus
IML	Inner Molecular Layer
PERK	Phosphorylated ERK1/2
Erα	Estrogen Receptor Alpha
HPH	Hypothalamic-pituitary-hippocampal Axis
GnRH	Gonadotropin-Releasing Hormone
LH	Luteinizing Hormone
FSH	Follicle-Stimulating Hormone
HPH	Hypothalamic-Pituitary-Hippocampus axis
ML	Molecular layer
GAPDH	Glyceraldehyde-3-phosphate dehydrogenase
SIRT1	Sirtuin 1
BDNF	Brain-derived neurotrophic factor
RAD	Stratum radiatum
